# Increase of Yield, Lycopene, and Lutein Content in Tomatoes Grown Under Continuous PAR Spectrum LED Lighting

**DOI:** 10.3389/fpls.2021.611236

**Published:** 2021-02-26

**Authors:** Dennis Dannehl, Thomas Schwend, Daniel Veit, Uwe Schmidt

**Affiliations:** ^1^Division Biosystems Engineering, Faculty of Life Sciences, Albrecht Daniel Thaer - Institute of Agricultural and Horticultural Sciences, Humboldt-Universität zu Berlin, Berlin, Germany; ^2^BLV Licht-und Vakuumtechnik GmbH, Steinhöring, Germany; ^3^Max Planck Institute for Chemical Ecology, Jena, Germany

**Keywords:** LED lighting, secondary metabolites, tomato, supplementary lighting, full spectrum LED, carotenoids, greenhouse

## Abstract

Light emitting diodes (LEDs) are an energy efficient alternative to high-pressure sodium (HPS) lighting in tomato cultivation. In the past years, we have learned a lot about the effect of red and blue LEDs on plant growth and yield of tomatoes. From previous studies, we know that plants absorb and utilize most of the visible spectrum for photosynthesis. This part of the spectrum is referred to as the photosynthetically active radiation (PAR). We designed a LED fixture with an emission spectrum that partially matches the range of 400 to 700 nm and thus partially covers the absorption spectrum of photosynthetic pigments in tomato leaves. Tomato plants grown under this fixture were significantly taller and produced a higher fruit yield (14%) than plants grown under HPS lighting. There was no difference in the number of leaves and trusses, leaf area, stem diameter, the electron transport rate, and the normalized difference vegetation index. Lycopene and lutein contents in tomatoes were 18% and 142% higher when they were exposed to the LED fixture. However, the ß-carotene content was not different between the light treatments. Transpiration rate under LED was significantly lower (40%), while the light use efficiency (LUE) was significantly higher (19%) compared to HPS lighting. These data show that an LED fixture with an emission spectrum covering the entire PAR range can improve LUE, yields, and content of secondary metabolites in tomatoes compared to HPS lighting.

## Introduction

Tomato is one of the most important greenhouse crops in the world. They are an important dietary source of carotenoids, a class of compounds that may have beneficial effects on human health, e.g., the reduction in the occurrence of inflammations and human prostate cancer (Kotake-Nara et al., 2001; Jacob et al., 2008). Tomatoes are nowadays also produced during winter season. We owe this to the development of supplementary greenhouse lightening. For the last 60 years high-pressure sodium (HPS) lamps were the working horse of the greenhouse industry due to their long operating life and low acquisition costs (Ouzounis et al., 2018). However, in the past years, light emitting diodes (LEDs) have become increasingly important as a more energy efficient alternative, in particular for tomato cultivation, which requires a daily light integral of up to 25 mol m^–2^ d^–1^ (Moe et al., 2005). Furthermore, in contrast to HPS lighting, which emits dominantly yellow and orange and very little blue light (∼5%) (Terfa et al., 2013), LED lighting can be designed to meet to photosynthesis action spectrum of the crop (Jou et al., 2015). The action spectrum of tomato leaves is well known (McCree, 1971). The photosynthesis action spectrum shows two peaks in the red and blue part of the visible spectrum that coincide with chlorophyll absorption. However, photosynthesis occurs over the entire range of wavelengths between 400 to 700 nm. The effect of these wavelengths is mediated by carotenoids, a diverse group of pigments, which are associated with the light harvesting complex (Frank and Cogdell, 1993; Hashimoto et al., 2016).

Although the role of carotenoids in photosynthesis is well appreciated, wavelengths other than red and blue had rarely been used in academic studies on the effect of LED lightings in tomato production. Consequently, there is a lot of information about the effect of red and blue light used as overhead-lighting, inter-lighting or hybrid-lighting (LEDs + HPS) on plant growth and yield of tomatoes (Dueck et al., 2011; Hogewoning et al., 2012; Gajc-Wolska et al., 2013; Gomez et al., 2013; Deram et al., 2014; Gómez and Mitchell, 2015; Tewolde et al., 2016; Gilli et al., 2018; Lanoue et al., 2019; Paponov et al., 2019). These studies showed that photosynthesis under a combination of red and blue light tends to be higher than under HPS lighting, but fruit yield is equal. It is not quite clear why there is no increase in fruit harvest under red and blue LEDs. Some studies also included additional far-red light (Hao et al., 2015; Zhang et al., 2019). Additional far-red light in combination with red LEDs and HPS appears to increase total fruit number. It has also been shown that the exposure to supplementary red and blue LED lighting increases the lycopene and ß-carotene content (Xie et al., 2019; Ngcobo et al., 2020).

To our best knowledge, only one research group studied the effects of spectrally optimized LED-lighting on algae growth (Ng et al., 2020). However, no information is available on the effects of continuous PAR spectrum LEDs on tomato growth and the tomato carotenoid content.

In order to study the effect of a continuous PAR spectrum on tomato growth and plant responses, such as light use efficiency, transpiration mass flow density and the concentrations of different carotenoids in tomatoes, a new LED fixture was designed. The emission spectrum of the fixture matches the waveband from 400 to 750 nm and in certain areas approximates the absorption spectrum of photosynthetic pigments in tomato leaves. This fixture was used to grow tomato plants in a greenhouse and compare the morphological and -physiological responses to plants grown under HPS lighting.

## Materials and Methods

### Supplementary Lighting

**FIGURE 1:**
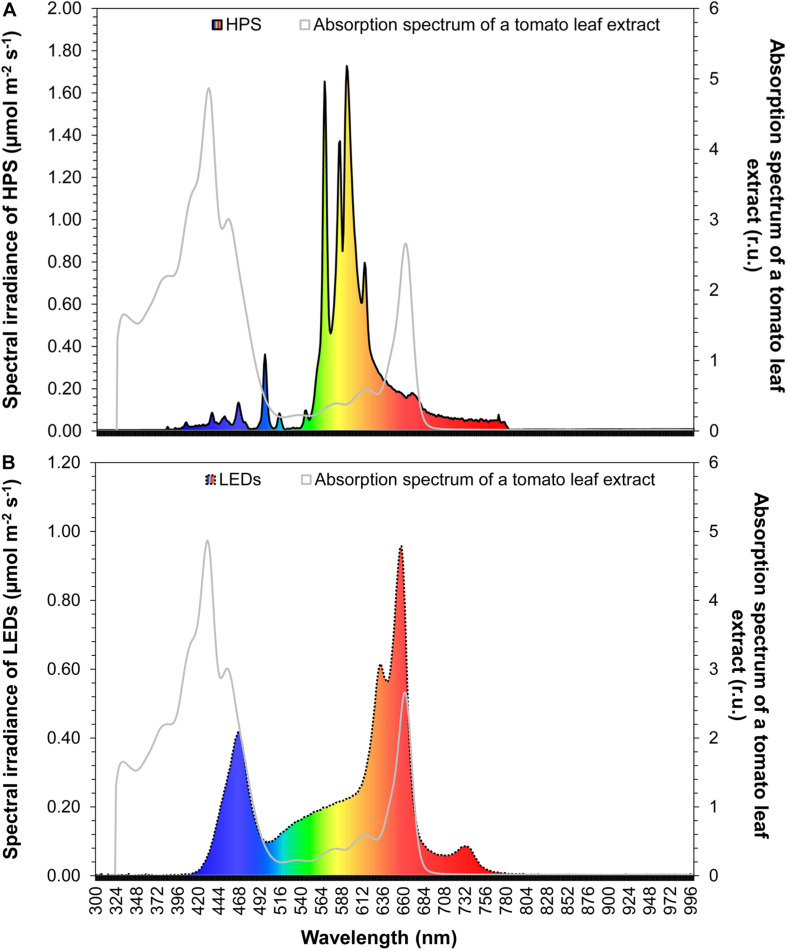
Comparison of the HPS **(A)** and LED **(B)** light spectrum with the absorption spectrum of a tomato leaf extract. Tomato leaves were extracted in acetone.

We designed a fixture with an emission spectrum that matches partially the PAR spectral range and in some parts approximates the absorption spectrum of photosynthetic pigments in tomato leaves (Figure 1). The leaf absorption spectrum was measured in acetone (Lichtenthaler and Wellburn, 1983). The LED fixture consisted of 14 LEDs (Roschwege GmbH, Greifenstein, Germany) combined with zoom lenses (B & M Optics GmBH, Limburg, Germany), which have a radiation angle of 60° to achieve an average light intensity of 55 μmol m^–2^s^–1^ at a distance of 1.8 m between the lamp and the bottom. The LED fixture consisted of three warm white LED’s (3000 K), 3 cool white LED’s (6000 K), two blue and red multichip LEDs (380 – 840 nm), 2 LED’s (630 nm), 3 LED’s (660 nm) and 1 LED (720 nm). Each LED had 10 W. The LED fixture had the following dimension: width = 20 cm, length = 50 cm, depth = 15 cm. This LED system was compared with a high-pressure sodium lamp (HPS) (SON-T Agro 400, Koninklijke Philips N.V., Amsterdam, Netherlands), which had 400 W. The HPS emission spectrum is displayed in Figure 1. In a distance of 1.8 m, the photosynthetic active radiation of the LED and HPS fixture was the same.

### Plant Material and Experimental Setup

Tomato plants (*Solanum lycopersicum* L., cv. Purezza) (Syngenta AG, Basel, Switzerland) were planted into a 75 m^2^ compartment of an experimental Venlo-type greenhouse at Humboldt-Universität zu Berlin. The seeds were germinated for 2 weeks in perlite (Perligran premium, Knauf Aquapanel GmbH, Dortmund, Germany) (October 11, 2018). The seedlings were transferred to 7.5 L containers filled with a horticultural substrate (substrate 1, Klasmann-Deilmann GmbH, Geeste, Germany). The plants were grown under the same conditions (temperature 23°C; relative humidity 80%, HPS lighting for 12 h from 6 am to 6 pm) until they reached a height of approximately 20 cm. This plant development stage was the starting point for the lighting experiments (November 22, 2018).

The experiments consisted of two light treatments, which were replicated three times. Each plot had the size of 1 m^2^ and contained five plants. The distance between plots was 3 m. The floor level heating was set at a target temperature of 23°C during the day and 18°C during the night. The ventilation was opened above 24°C. During the experiments, the temperature did not fall below the set night temperature. The maximum daytime temperature was reached in April and was 26.7°C. The energy screen was closed if the global radiation was below 3 W m^–2^. Plants were fertilized when required. In detail, the substrate moisture was measured with an analog tensiometer (Tensiometer, Step Systems GmbH, Nürnberg, Germany). If a suction tension of 120 hPa in the substrate was exceeded, watering was carried out until the nutrient solution ran out of the pot. The nutrient solution was prepared according to the protocol of Göhler and Molitor (2002). Supplementary LED and HPS lighting were used between 6.00 am and midnight if the solar photosynthetic photon flux density (PPFD) was below 360 μmol m^–2^ s^–1^.

### Assessment of Plant Growth and Yield

The average plant height, internode distance, stem diameter, number of trusses and leaves, as well as the leaf area were recorded during the first six weeks of cultivation. The number of leaves and trusses were counted, and all other parameters were measured with normal tools like a folding ruler and a caliper. Leaf area was calculated according to Dannehl et al. (2015). Leaf area per plant (m^2^ plant^–1^) is the sum of individual leaves. The stem diameter was measured at a height of 10 cm above ground. Tomatoes were harvested at ripening stage 10 (according to the Organisation for Economic Co-operation and Development, OECD colour gauge), counted and weighed. As such, the weight of each fruit was categorized according to different weight classes: marketable fruit > 50 g and non-marketable fruit ≤ 50 g. The end of cultivation was April 7, 2019.

### Light Use Efficiency, Transpiration Mass Flow Density, and Leaf Temperature

The light use efficiency (LUE) and transpiration mass flow density caused by both light treatments were measured using two gas exchange systems (BERMONIS, Steinbeis GmbH & Co. KG, Stuttgart, Germany) and recorded all 30 s. Each gas exchange system consisted of 10 leaf chambers. As such, 10 leaf chambers were distributed on five plants of each light treatment. One leaf chamber was attached to the first and one to the second fully developed leaf of each randomly selected plant. Simultaneously, PPFD was measured (Li-190R, LICOR, Lincoln, NE, United States) at each leaf chamber. This was done three times between 8.00 pm and midnight for a period of 1 week, starting on 6 February. LUE is here defined as CO_2_ uptake (μmol CO_2_ m^–2^ s^–1^) divided by the incident PPFD (μmol m^–2^ s^–1^) and expressed as μmol CO_2_ μmol^–1^ PPFD. The transpiration mass flow density was measured with the same experimental setup using the BERMONIS system and expressed as mg m^–2^ s^–1^.

Leaf temperatures were measured under both light treatments with thermoelements during five consecutive weeks (starting point 16 weeks after sowing), where mean values were calculated per week. Five thermoelements were fixed on the first fully developed leaf of five different plants.

### Optical Readings

A portable, hand-held spectrophotometer (Pigment Analyzer PA-1101/801, Control in Applied Physiology GbR, Falkensee, Germany) equipped with photodiode arrays and a NIR spectrometer (MMS1 NIR enh., Carl Zeiss, Germany) were used to measure the reflectance spectra of tomato leaves in the visible and near infrared range between 402 and 1048 nm with a spectral resolution of 3.3 nm. An integrated light cup equipped with LEDs, capturing the entire recorded wavelength range, served as the light source. Spectralon (20% certified, Labsphere Ltd., North Sutton, NH, United States) was used as the white reference to calibrate this device. During three consecutive weeks (starting point 16 weeks after sowing), five measurements were taken from the first fully developed leaf of every plant in the plot, and the average reflectance indices were used for the estimation of the normalized difference vegetation index (NDVI). The NDVI was calculated as NDVI = (*I*_NIR_-*I*_Red_)/(*I*_NIR_ + *I*_Red_); we used *I*_750_ for *I*_NIR_ and *I*_680_ for *I*_Red_ as described by Richardson et al. (2002). At the same measuring dates as mentioned in NDVI measurements, nine randomly selected leaflets per light treatment group were used to measure the electron-transport rate (ETR). This was measured non-destructively using a pulse-amplitude modulated device (IMAGING MAXI-PAM, M-series, Heinz Walz GmbH, Eltrich, Germany) on dark adapted leaflets (20 min). Among others, this method was used to study the photosynthetic performance of plants. Based on these data, conclusions on plant health or stress factors can be drawn (Baker, 2008). The ETR was calculated as follows: ETR = Y (II) x PPFD x 0.84 × 0.5. In this context, Y (II) is defined as quantum efficiency of photosystem two (PS II). The factor 0.84 derives from the assumption that 84% of the incident PPFD is absorbed by the leaves. The factor 0.5 indicates the average energy distribution between PS I and PS II (Stemke and Santiago, 2011).

### Chemical Analysis

To measure the carotenoid concentrations of tomatoes, 15 tomatoes (>70 g) were harvested randomly from different plants in each plot at ripening stage 9. This sampling was repeated three times in three consecutive weeks, starting 22 weeks after sowing. The harvested tomatoes were homogenized with a blender (Kenwood HB856, De’Longhi Deutschland GmbH, Neu-Isenburg, Germany). Lycopene, ß-carotene, and lutein were extracted using the method of Fish et al. (2002). Afterward, the extracts were measured spectrophotometrically in transmission geometry in the range from 350 to 850 nm in a resolution of 1 nm (Lambda 950; Perkin-Elmer, United States). These data were used to calculate the lycopene, ß-carotene, and lutein concentrations using iterative multiple linear regressions (iMLRs), a method developed by Pflanz and Zude (2008).

### Data Analysis

All data represent mean values and standard deviations. The normal distribution of the data was tested using the Kolmogorov–Smirnov test. These tests were followed by student’s *t*-tests to calculate significant differences at a significant level of *p* ≤ 0.05. Standard deviations are illustrated by ± in tables and bars in figures. Significant differences are displayed with different small letters. All statistical tests were calculated using SPSS, package version 26.0.

## Results

### Duration of Artificial Light Exposure

Supplementary lighting was provided for 420 h in January, 412 h in February, 371 h in March, and 250 h in April. The ratio of daylight to supplementary lighting exposure changed as follows: 1:3 in January, 1:4 in February, 1:2 in March, and 1:1 in April.

### Plant Growth and Yield

Plants grown under LED lighting became significantly taller compared to those grown under HPS lighting eight weeks after sowing (Table 1). The difference in plant height was 6.5 cm two weeks after the beginning of the experiment and 11 cm by the end. The internodes of plants grown under LEDs were on average 1 cm longer, but the stems were equally thick (Table 1).

**TABLE 1:** Effects of different supplementary lightings on plant development and yield of tomato plants.

			Weeks after sowing					
Plant parameter	Unit	Light treatment	6	7	8	9	10	11
Plant height	cm	LEDs	24.1 ± 1.9a	51.0 ± 4.1a	82.1 ± 5.3b	112.4 ± 9.1b	143.4 ± 10.9b	172.1 ± 9.2b
		HPS	24.2 ± 1.6a	48.5 ± 4.1a	75.6 ± 5.8a	104.5 ± 8.5a	131.9 ± 7.9a	161.1 ± 6.3a
Internode distance	cm	LEDs	3.5 ± 0.3a	7.3 ± 0.7b	8.7 ± 0.7b	9.4 ± 0.9b	9.5 ± 0.8b	9.8 ± 0.6b
		HPS	3.2 ± 0.4a	6.2 ± 0.7a	7.7 ± 0.6a	8.4 ± 0.7a	8.5 ± 0.3a	8.7 ± 0.4a
Stem diameter	cm	LEDs	6.3 ± 0.4a	7.4 ± 0.3a	7.8 ± 0.5a	8.5 ± 0.4a	8.9 ± 0.6a	9.5 ± 0.5a
		HPS	6.2 ± 0.5a	7.2 ± 0.2a	7.7 ± 0.3a	8.4 ± 0.5a	8.9 ± 0.3a	9.3 ± 0.6a
Trusses	number plant^–1^	LEDs	0.0 ± 0.0a	0.0 ± 0.0a	0.0 ± 0.0a	1.0 ± 0.0a	2.0 ± 0.0a	3.1 ± 0.3a
		HPS	0.0 ± 0.0a	0.0 ± 0.0a	0.0 ± 0.0a	1.0 ± 0.0a	2.0 ± 0.0a	3.1 ± 0.3a
Leaves	number plant^–1^	LEDs	3.8 ± 0.4a	5.1 ± 0.3a	7.6 ± 0.5a	9.9 ± 0.6a	13.4 ± 0.5a	15.4 ± 0.9a
		HPS	3.8 ± 0.3a	5.1 ± 0.3a	7.8 ± 0.4a	10.2 ± 0.4a	13.6 ± 0.5a	16.0 ± 0.5a
Leafarea	cm^2^ plant^–1^	LEDs	489.3 ± 60.9a	965.1 ± 99.9a	1713.0 ± 132.7a	2872.0 ± 186.0a	4946.0 ± 351.3a	6674.9 ± 431.5a
		HPS	509.6 ± 52.1a	975.1 ± 90.6a	1863.4 ± 124.5b	3243.6 ± 228.4b	5346.8 ± 482.9a	7196.3 ± 589.2a

			**End of the experiments**					

Yield	g plant^–1^	LEDs			2102.1 ± 159.0b			
		HPS			1844.3 ± 91.8a			
Fruit weight	g fruit^–1^	LEDs			96.7 ± 5.2a			
		HPS			92.4 ± 5.4a			

*Values represent means of three plots per light treatment (*n* = 3) ± standard deviation. Values followed by the same letter do not differ significantly according to student’s *t*-tests (*p* < 0.05).*

There was no significant difference in the number of leaves and leaf area per plant, except for a short period at the beginning of growth in weeks 8 and 9 after sowing, when the leaf area of plants exposed to LED light was smaller. Despite having the same number of trusses as plants grown under HPS, marketable yield of plants grown under LED lighting was significantly higher (by 257.8 gram per plant) compared to that caused by HPS lighting. Non-marketable fruits were not found for both light treatments. The average fruit weight was also higher under LED lighting (Table 1).

### Effects on Leaf Temperature, LUE, Transpiration Mass Flow Density, ETR, and NDVI

As plants grew toward the supplementary lighting, leaf temperatures increased from 19.9°C to 21.7°C under LED lighting and from 20.7°C to 23.2°C under HPS lighting (Table 2). The leaf temperature of plants exposed to LED light was always significantly lower, 0.8°C at the beginning and 1.5°C at the end of measurements (Table 2).

**TABLE 2:** Leaf temperature (°C) under LED and HPS lighting.

	Weeks after sowing				
Light treatment	16	17	18	19	20
LEDs	19.9 ± 0.1	20.8 ± 0.2	21.2 ± 0.1	21.3 ± 0.2	21.7 ± 0.3
HPS	20.7 ± 0.1	21.8 ± 0.5	22.2 ± 0.2	22.5 ± 0.5	23.2 ± 0.8

*Data represent mean values of five repetitions in five consecutive weeks (*n* = 5). Differences in leaf temperature were evaluated using student’s *t-*tests. Different small letters illustrate significant differences (*p* < 0.05).*

**FIGURE 2:**
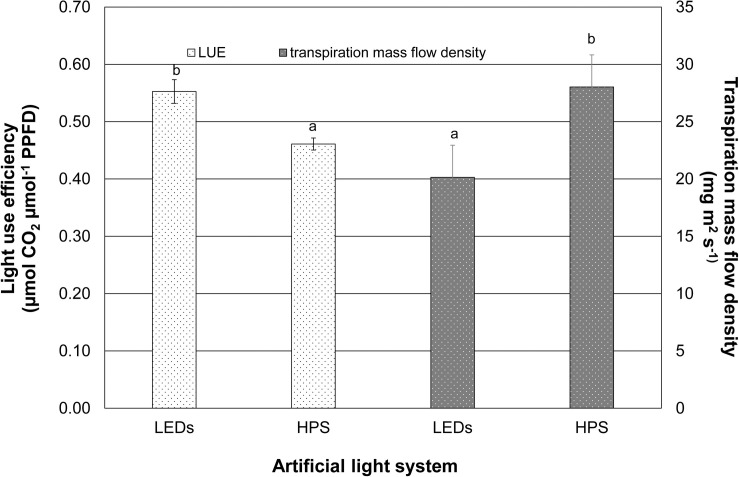
Light use efficiency and transpiration mass flow density caused by LEDs and HPS lighting. Data represent mean values of three repetitions (*n* = 3). Light use efficiency and transpiration mass flow density were tested using student’s *t*-tests. Different small letters indicate significant differences (*p* < 0.05).

Figure 2 shows the average LUE of the supplementary lighting without daylight during a period of 3 weeks. Although the PPFD was identical from both lighting systems, the LED fixture significantly increased the light use efficiency of the plants by 20% compared to those that grew under the HPS lighting. In contrast, the transpiration mass flow density of the tomato plants under LED exposure (20.15 mg m^2^ s^–1^) was lower than that of the tomato plants under HPS exposure (28.03 mg m^2^ s^–1^). A significantly higher transpiration mass flow density (by 39%) caused by HPS lighting was calculated (Figure 2).

**FIGURE 3:**
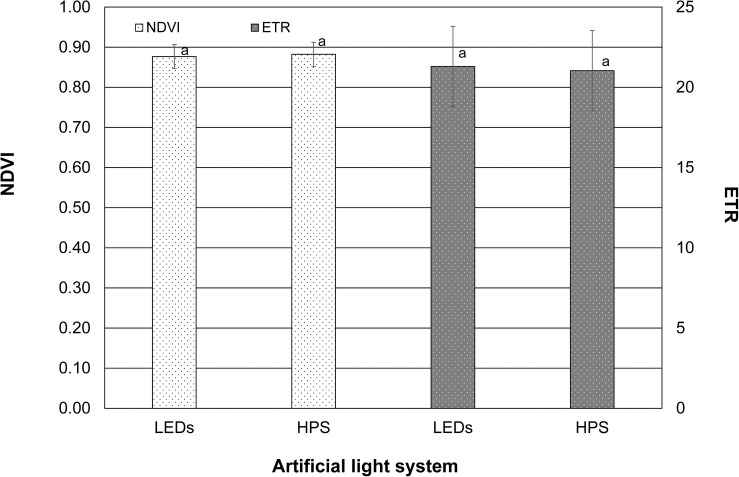
Normalized difference vegetation index (NDVI) and electron transport rate (ETR) of tomato leaves exposed to LEDs and HPS lighting. Data represent mean values of three repetitions in three consecutive weeks (*n* = 3). NDVI and ETR were tested using student’s *t*-tests. Different small letters illustrate significant differences (*p* < 0.05).

Neither LEDs nor HPS lighting led to a significant change in the values regarding the calculated electron transport rate and NDVI. The values of both parameters were almost identical and were 0.9 and 21.0, respectively (Figure 3).

### Effects on Carotenoids

There were significant differences in the carotenoid concentrations between the light treatments. The lycopene concentration of tomatoes grown under LED lighting was 18% higher (910.3 versus 773.8 μg lycopene g^–1^ DW) and the lutein concentration was more than two times higher (24.7 versus 10.2 μg lutein g^–1^ DW) (Table 3). The ß-carotene concentrations of tomatoes, on the other hand, were similar (44.4 versus 46.2 μg g^–1^ DW).

**TABLE 3:** Influence of LEDs and HPS supplementary lighting on carotenoid concentration in tomatoes.

	Carotenoid concentration in tomatoes (μg g^–1^ DW)		
Light treatment	Lycopene	ß-carotene	Lutein
LEDs	910.3 ± 53.3b	44.4 ± 3.0a	24.7 ± 3.3b
HPS	773.8 ± 45.1a	46.2 ± 3.5a	10.2 ± 3.0a

*Values represent means of three repetitions per light treatment in three consecutive weeks (*n* = 3) ± standard deviation. Values followed by the same letter do not differ significantly according to student’s *t-*tests (*p* < 0.05).*

## Discussion

### Plant Growth and Yield Under Different Supplementary Lightings

In this article, we provide evidence that LED lighting with an emission spectrum that partially matches the range of 400 to 700 nm can accelerate plant growth and increase the yield of tomatoes. Similar results in terms of the stem development were demonstrated for cucumbers, sweet basil, and tomatoes when a combination of blue, green, and red LEDs (Särkkä et al., 2017); blue, yellow, and red LEDs (Carvalho et al., 2016); or only green LEDs combined with daylight (Snowden et al., 2016) was used, respectively. Our yield data, however, are different to others, who have reported that a LED lighting with an emission spectrum that is optimized for chlorophyll absorption (blue and red) produces similar or even less yield than HPS lighting (Dueck et al., 2011; Gomez et al., 2013; Fanwoua et al., 2019). Although the cultivation conditions in these studies differ substantially from ours (most used interlight, different cultivar), our data suggest that a full spectrum offers advantages over a simple red and blue emission spectrum in tomato plants. Evidence that more complex spectra can increase tomato yield was also provided by Kim et al. (2019), who showed that adding far-red to red and blue increases yield. Kim et al. (2019) attributed the effect of far-red light to an increase in photosynthesis, an effect that is well known in the literature as Emerson effect (Emerson, 1957). An over-proportional increase in photosynthesis due to changes in the spectrum, like for instance the addition of far-red to red light, results in an increase in light use efficiency. In our study, we found that the light use efficiency under a continuous PAR spectrum LED lighting was higher than under HPS lighting, indicating that the additional wavelengths are not only absorbed but actively used for photosynthesis. This conclusion is in agreement with Yang et al. (2016), who found that the addition of yellow light to blue and red light can greatly enhance the LUE of purple cabbage. The LUE can be affected by plant stress. For instance, water deficiency, photoinhibition, or low light quality can reduce photosynthesis despite a sufficiently high PPFD. An indicator for plant stress is ETR. ETR is reduced when plants suffer from water stress (Ögren and Öquist, 1985) or exposure to monochromatic light (Wang et al., 2009; Xiaoying et al., 2012). More complex spectra have higher ETR (Xiaoying et al., 2012). Another indicator is NDVI. NDVI increases when the chlorophyll concentration in a canopy increases (Yoder and Waring, 1994; Gitelson et al., 2003). Therefore, NDVI can be used to monitor vitality, especially senescence of leaves. In this study, we did not find differences in NDVI or ETR. Therefore, we can exclude that the plants were stressed. These data support our conclusion that the LUE is higher under the LED lighting because the wavelengths are effectively used for photosynthesis. In summary, the data presented here show that the absorption of the additional wavelengths of the continuous PAR spectrum LED leads to higher photosynthesis and consequently longer plants and higher fruit yield.

Photosynthesis is more than photon absorption. For instance, gas exchange at the stomata and RuBisCo activity are of vital importance to photosynthesis. The latter increases with higher leaf temperatures. HPS lighting emits ample infra-red radiation, which is not captured by the photosystem but increases leaf temperature instead (Nelson and Bugbee, 2015). Infra-red radiation can have a substantial effect on photosynthesis and yield (Hernández and Kubota, 2015; Bergstrand et al., 2016). In our experiment, we measured the expected 1.5°C higher leaf temperature under HPS, and as a consequence, a higher transpiration rate was also found in other studies (Dueck et al., 2011; Gajc-Wolska et al., 2013; Kim et al., 2019). Sage and Sharkey (1987) had shown that the rate of carbon dioxide uptake (e.g., photosynthesis) increased with increasing temperatures, until it reaches a critical point, after which it rapidly decreases. The critical point of tomatoes is between 25°C and 27°C. In our study, the leaf temperature under HPS lighting was higher than under LEDs, but lower than 25°C. One, therefore, can expect that the activity of the photosystem, the Calvin cycle activity, and the gas exchange are higher under HPS. However, it appears that the temperature advantage is not sufficient to compensate for the effects of the LED spectrum.

Since there is no evidence that the LED spectrum promoted photosynthesis downstream of the photosystem, via control of stomata opening for instance, it is most likely that the additional wavelengths are effectively used for nicotinamide adenine dinucleotide phosphate reduction.

High photosynthesis is a prerequisite but not a guarantee for high yield. There is sometimes surprisingly little correlation between photosynthesis measured at a single leaf and total plant biomass or fruit yield. Therefore, horticulturists prefer to use the leaf area index to model and predict plant growth (Heuvelink et al., 2004). The leaf area depends on a variety of parameters, of which light spectral quality is one. Red light tends to increase leaf area, while blue light tends to decrease it (Bantis et al., 2018). These effects are not mediated by photosynthesis, but by photoreceptors (Kami et al., 2010). However, in our study there was no difference in leaf area despite a significant difference in tomato yield. Therefore, these data indicate that the effect of the spectrum is not mediated by alternative pathways (e.g., photoreceptors) but via the photosystem.

We know from other studies that effects of the spectra on photosynthesis can vary between species and even between varieties (Ouzounis et al., 2016). It is at the moment not clear how this diversity will be handled in the future. Perhaps, one way is to find common responses on a physiological or molecular level that allow the prediction of plant responses.

### Effects on Carotenoids of Tomatoes

Generally, the concentrations of carotenoids in tomatoes were consistent with those reported in literature (Frusciante et al., 2007). The biosynthesis of lycopene and lutein was affected by the different light treatments. The lycopene and lutein concentrations of tomatoes grown under LED lighting were 18 and 142%, respectively, higher than that grown under HPS lighting (Table 3). The increase in lycopene of tomatoes caused by a combination of blue and red LEDs was also demonstrated by Ngcobo et al. (2020). They also found that these LEDs caused a higher concentration of ß-carotene than a combination of yellow and blue or red LEDs, while the ß-carotene concentration in the present study did not change when illuminated with LEDs or HPS lighting. The regulation of carotenoid biosynthesis in ripening tomato fruits has been extensively studied (Bramley, 2002). In green tissue, lycopene is further converted to ß-carotene by lycopene cyclases. Therefore, ß-carotene and lutein levels increase during the first days of fruits ripening, long before lycopene appears (Ntagkas et al., 2020). However, during the later stage of tomato fruit ripening, the mRNA of the lycopene ß- and ε-cyclase enzymes are downregulated (Pecker et al., 1996; Ronen et al., 1999). As a consequence, lycopene accumulates, while the ß-carotene level starts decreasing. In contrast to the intermediate product ß-carotene, lutein levels stay constant after the metabolism is shifted to lycopene production. One, therefore, can assume that the differences in lutein content manifest before the fruits ripen, while differences in ß-carotene levels are a combination of early formation and later conversion. Aherne et al. (2009), furthermore, showed that the geographical origin has a strong influence on the ß-carotene concentration and that this influence is higher than that of the variety. This study indicates that cultivation and environmental conditions, such as light intensity, light spectrum, and fruit temperature, affect the regulation of the ß-carotene biosynthesis pathway in tomatoes.

In a previous article, we have shown that tomatoes grown in optimized climate conditions increase photosynthesis and ß-carotene concentration in fruit (Dannehl et al., 2014). These data indicate that photosynthesis provides the molecular precursors for the biosynthesis of ß-carotene in fruit and subsequently lutein and lycopene. It is therefore reasonable to assume that the increase of lycopene and lutein concentrations in this study is driven by a higher LUE caused by the continuous PAR spectrum LED-fixture. Beside photosynthesis, there is ample evidence that lycopene biosynthesis in tomatoes is also regulated by photoreceptors. Alba et al. (2000) reported that red light promotes and far-red light reduces lycopene biosynthesis and that this effect is mediated by photochromes. Xie et al. (2019) have analyzed tomatoes exposed to blue and red LEDs on a gene expression level using qRT-PCR. They found out that blue and red light increased the lycopene concentration in tomatoes by inducing light receptors that modulate phytochrome-interacting factors and ELONGATED HYPOCOTYL 5 activations to mediate phytoene synthase 1 (*PSY*). *PSY* is the main enzyme in the carotenoid pathway (Bramley, 2002). Furthermore, they found that blue light showed a much stronger effect than red light. Li and Kubota (2009) and Ouzounis et al. (2015) also showed that the concentration of carotenoids in green lettuce increased when blue light was supplemented. Ntagkas et al. (2020), on the other hand, showed that the spectrum has no effect on the lutein concentration. Blue light appears to promote the lutein content more than red light for instance. However, the data are insufficient to explain the increase in lutein in our experiment. It is therefore possible that the higher proportion of blue light, and perhaps a lower percentage of far-red light, emitted by our LED fixture had an additional effect on the lycopene and lutein concentrations, but we think that the main effect is an increase in LUE. As to why the ß-carotene concentration is constant while the other carotenes increase, our data do not provide a simple explanation. Ntagkas et al. (2020) showed that zeaxanthin, the product of ß-carotene conversion, increases in tomato fruits under blue and white light. These data indicate that ß-carotene is further converted under white light. Therefore, it is likely that the initially higher ß-carotene levels under white light were later converted to zeaxanthin. At a later stage of fruit development zeaxanthin levels in tomato fruits also drop rapidly, indicating that either the conversion is inhibited or the degradation accelerated. These findings may indicate that ß-carotene levels reach a steady-state toward the end of fruit ripening. However, at the moment, there are no data supporting this hypothesis.

Beside light, it is also known that an increase in mean ambient temperature from 18°C to 22°C favors the accumulation of carotenoids in tomatoes (Krumbein et al., 2006). Since the leaf temperature was increased under the influence of HPS lighting, it can be assumed that the fruit temperature was also increased. However, Krumbein’s study also shows that a temperature increase of 2°C has no effect. Since we only measured a maximum temperature increase of 1.5°C regarding the leaf temperature, the temperature influence on the carotenoid accumulation can be neglected.

## Conclusion

In this article, we show that LED with an emission spectrum that partially matches the PAR range is more effective than HPS supplementary lighting. Based on our results, it can be concluded that our LED fixture positively affected plant growth and LUE, increasing the yield and concentrations of lycopene and lutein. In comparison to HPS lighting, the LED spectrum reduced the transpiration mass flow density, while keeping the electron transport rate and the normalized difference vegetation index at the same level.

In recent years, white LEDs have become more and more efficient, so that more manufacturers have begun to incorporate them into their fixtures. Our data clearly show that from a horticultural point of view, the use of continuous PAR spectrum LEDs can be considered as a useful tool, as not only the yield but also the carotenoid concentration in tomatoes can be improved.

## Data Availability Statement

The original contributions presented in the study are included in the article/supplementary material, further inquiries can be directed to the corresponding author/s.

## Author Contributions

DD brought up the idea, contributed to the study conception and design, acquisition, statistical analysis, and interpretation of data, and writing of the manuscript, and discussed with the reviewers and editors to modify the manuscript. TS made recommendations and suggestions and discussed with the reviewers and editor to modify the manuscript. DV manufactured the full spectrum LED fixture. US made recommendations and modified the manuscript. All authors contributed to the article and approved the submitted version.

## Conflict of Interest

TS was employed by BLV Licht-und Vakuumtechnik GmbH. The remaining authors declare that the research was conducted in the absence of any commercial or financial relationships that could be conducted as a potential conflict of interest.
